# Effect of conventional cigarettes and e-cigarettes on salivary biomarkers: A systematic review

**DOI:** 10.34172/japid.2024.006

**Published:** 2024-04-22

**Authors:** Amirmohammad Dolatabadi, Faranak Noori, Amir Raee

**Affiliations:** ^1^Department of Periodontology, School of Dentistry, Tehran University of Medical Sciences, Tehran, Iran; ^2^Department of Endodontics, School of Dentistry, Tehran University of Medical Sciences, Tehran, Iran

**Keywords:** Cytokines, Smokers, Tobacco products

## Abstract

**Background.:**

E-cigarette consumption is increasing, and like conventional smoking, it can cause some harmful effects. This systematic review compared the effect of conventional cigarettes and e-cigarettes on salivary biomarkers.

**Methods.:**

The search strategies included electronic databases (Medline/PubMed, Scopus, EMBASE) and related journals up to May 2023. A qualitative assessment was performed on data extracted from the included studies. Seven studies were included in this systematic review (number of patients=563).

**Results.:**

Eleven biomarkers were assessed and compared between e-cigarette and conventional cigarette smokers. The data showed that the different effects of electronic and conventional cigarettes on the level of these biomarkers were not achievable. Due to the heterogeneity of the studies (I^2^ statistic>90%), performing a meta-analysis was impossible. Even after a sub-group classification, homogeneous data were not achieved.

**Conclusion.:**

The current data do not provide evidence of obtainable outcomes between conventional cigarettes and e-cigarettes on salivary biomarkers.

## Introduction

 Smoking has harmful effects on human health that have been discussed in several studies.^1^ Traditional tobacco products (e.g., smoking combustible cigarettes) can increase the risk of severe disorders like cancer and cardiopulmonary and metabolic diseases.^2^ Smoking also has significant adverse effects on oral health, with relationships between smoking and periodontal diseases, wound healing, and oral cancers.^1^

 Electronic cigarettes have become popular, with over two million Britons now regularly vaping.^3^ Despite the existence of the idea that e-cig vaping is safer than cigarette smoking, many epidemiological studies have shown its adverse effects.^4^ In e-cigarettes, nicotine is provided for inhalation by heating a solution that contains water, nicotine, propylene glycol, and vegetable glycerin.^3^ Recent studies have shown that e-cigs can change heart rate, blood pressure, and other vital signs and symptoms. Smoking e-cigarettes can increase neutrophil activation and change mucin secretion. Because of the exposure to harmful organic and inorganic compounds (including metals), e-cigarette users are more susceptible to developing cancer than nonusers.^4^

 It has been reported that periodontal status, plaque index (PI), clinical attachment loss (CAL), probing depth (PD), and marginal bone loss are worse in individuals using e-cigarettes and other electronic nicotine delivery systems (ENDS) than in the controls (individuals who have never used tobacco in any form).^5^

 Different biofluids, such as blood, gingival crevicular fluid, and saliva, have been used for their diagnostic or prognostic value for disease detection.^6^ Considering its advantages, such as ease and noninvasive collection, saliva can be a potential alternative to blood tests. Also, in many studies, saliva has been used as a target vehicle for different biomarkers in oral diseases.^7^

 Various salivary biomarkers can play important roles in oral health status. For instance, interleukin-6 (IL-6) can activate osteoclast formation and facilitate bone resorption and T-cell differentiation. In addition, IL-6 is implicated in periodontitis. Another crucial biomarker to indicate is the IL-8, which is involved in the selective recruitment and activation of neutrophils.^3^ In addition, the existence of many biomarkers in saliva causes a benefit in diagnostic and prognostic issues. For instance, salivary levels of tumor necrosis factor α (TNF-α), IL-1, IL-4, IL-6, and IL-8 have been described as relevant biomarkers for oral lichen planus diagnosis and prognosis.^8^ Also, IL-1β, TNF-α, IL-6, and the receptor activator of nuclear factor κB ligand (RANKL), among other cytokines, are known to be involved in immune response regulation in periodontal diseases.^9^

 Due to the abovementioned features of saliva, it is a favorable oral fluid to determine the health status of the oral cavity, including the presence of periodontal diseases.^10^

 To the best of our knowledge, no systematic review has been conducted on the in vivo effects of conventional cigarettes and e-cigarettes on salivary biomarkers. Therefore, the current study compared the effects of conventional cigarettes and e-cigarettes on salivary biomarkers.

## Methods

 The present systematic review was conducted according to PRISMA statement guidelines.^11^ The protocol of this review was registered in the International Prospective Register of Systematic Reviews (PROSPERO) with the registration number CRD42023440189. The question focused on in this study was: “What is the comparative effect of conventional cigarettes and e-cigarettes on salivary biomarkers?” The differences in salivary biomarkers in conventional and e-cigarette smokers were considered the primary outcome of this systematic review.

 This question has been articulated as follows:

- Population: electronic and conventional cigarette smokers
- Intervention: conventional cigarette smokers
- Comparison: electronic cigarette smoking
- Outcomes: salivary biomarkers

###  Search strategy

 We systematically reviewed the literature within three main electronic databases (Medline/PubMed, Scopus, and EMBASE) to identify all articles comparing salivary biomarkers between conventional and e-cigarette smokers up to May 2023. We also searched cross-references to complement the evidence given in this review. The literature was searched using the electronic search strategy (Supplementary file 1).

 The present review included case-control and cross-sectional studies that compared salivary biomarkers in conventional and e-cigarette smokers. Retrospective studies, case series, case reports, animal studies, in vitro studies, letters, conference abstracts, and brief reports were excluded.

###  Study selection

 Two authors (AD and AR) independently screened the titles (and abstracts, if necessary) of the studies to determine the articles that met the inclusion criteria. If there was any conflict, a third reviewer (FN) made a judgment. All full texts of the studies meeting the inclusion criteria were assessed for quality.

###  Quality assessment

 Two reviewers (AD and AR) independently assessed the quality of the included studies. For each study, the risk of bias was assessed using The Joanna Briggs Institute’s Risk of Bias tool. The tool comprises eight items (clarity of criteria for inclusion, description of e the study subjects and the setting, validity and reliability of exposure measurement, using objective, standard criteria for measurement of the condition, identification of confounding factors, strategies to deal with confounding factors, and validity and reliability of outcomes measurement, using appropriate statistical analysis). Assessing bias led to the judgment of low risk of bias if all the domains were evaluated as low risk of bias, unclear risk of bias if at least one item was assessed as unclear risk of bias, or high risk of bias if at least one item was rated as high risk of bias. Any disagreement was resolved by discussion with a third reviewer (FN) to reach a consensus (Table 1).

**Table 1:** Quality assessment of included studies

**Study**	**Were the criteria for inclusion in the sample clearly defined?**	**Were the study subjects and the setting described in detail?**	**Was the exposure measured in a valid and reliable way?**	**Were objective, standard criteria used for measurement of the condition?**	**Were confounding factors identified?**	**Were strategies to deal with confounding factors stated?**	**Were the outcomes measured in a valid and reliable way?**	**Was appropriate statistical analysis used?**
Ye et al,^12^ 2018	Yes	Yes	Yes	Yes	No	No	Yes	Yes
Mokeem et al,^13^ 2018	Yes	Yes	Yes	Yes	No	No	Yes	Yes
Verma et al,^15^ 2021	Yes	Yes	Yes	Yes	No	No	Yes	Unclear
Faridoun et al,^17^ 2021	No	Yes	Yes	Yes	No	Unclear	Yes	Yes
Ali et al,^5^ 2022	Yes	Yes	Yes	Yes	YES	Unclear	Yes	Yes
Kamal et al,^14^ 2022	Yes	Yes	Yes	Yes	No	No	Yes	Yes
Miluna et al,^16^ 2022	Yes	Yes	Yes	Yes	No	Unclear	Yes	Yes

###  Data analysis

 The biomarkers’ level as a continuous outcome was presented as mean differences. All the outcomes were reported with their associated 95% confidence interval and analyzed in RevMan version 5.4 according to a random-effects model using the inverse-variance method for continuous outcomes. The heterogeneity of effects was evaluated using Higgins’ I^2^ statistic.

## Results

###  Study selection

**Figure 1:**
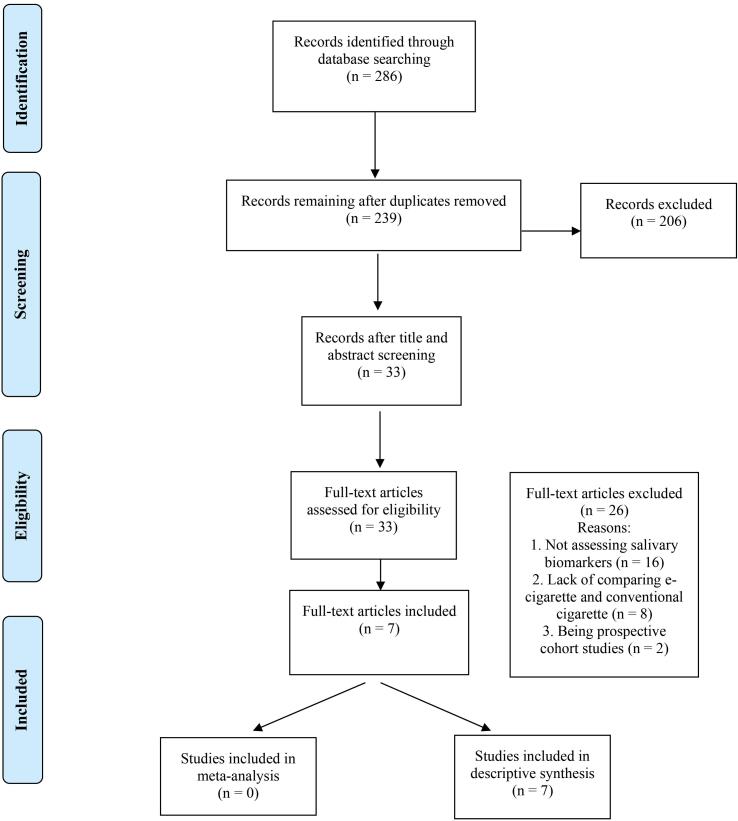
Flowchart of the Search strategy

 The search yielded 286 articles: 198 obtained via PubMed, 25 via Embase, 63 via Scopus, and 0 via hand research. After removing duplicates, 239 records were screened for titles and abstracts, and 206 studies were excluded due to not meeting the inclusion criteria, leaving 33 articles for full-text assessment. After a full-text review, 26 articles were excluded for the following reasons: not assessing salivary biomarkers, lack of comparison between e-cigarettes and conventional cigarettes, and being prospective cohort studies. Therefore, seven studies, all of which were case-control and cross-sectional, were included in this systematic review and used for the qualitative and quantitative analyses (see Figure 1).

Table 2 summarizes the characteristics of the studies included in this systematic review.

**Table 2:** Characteristics of the included studies

**Study**	**Study design**	**Outcome measures**	**Study groups**	**Population**	**Duration of smoking**
Verma et al^15^	Cross-sectional	IL1β, IL6, IL8, IL10, IL1RA, CRP, TNFα	e-cigarette smoker - conventional smoker - both smoker - nonsmoker	38 Males 22 Females	Not mentioned
Ye et al^12^	Cross-sectional	PGE-2, IL-1β	e-cigarette smoker - conventional smoker - both smoker - nonsmoker	24 Males 24 Females	Not mentioned
Miluna et al^16^	Cross-sectional	IL-6, IL-1β, IL-8, TNFα	Snus - Cigarettes - E-cigarettes - nonsmoker	38 Males 38 Females	Not mentioned
Mokeem et al^13^	Cross-sectional	IL-1β, IL-6	cigarette-smokers, waterpipe-smokers, E-cig users - never-smokers	154 Males	Cigarette: 16.2 ± 2.5 per day e-Cigarette: 9.2 ± 1.4 per day
Faridoun et al^17^	Cross-sectional	IL1β, IL6, IL8, IL10, IL1RA, CRP, TNFα	Conventional cigarettes - E-cigarettes - Mixed use - No smoking	37 Males 27 Females	Not mentioned
Ali et al^5^	Cross-sectional	IL-15, IL-18	Current cigarette smokers - ENDS users - Never-smokers with periodontitis - Never-smokers without periodontitis	54 Males 21 Females	Cigarette: 24.3 ± 0.7 pack years e-Cigarette: 12.5 ± 0.8 years
Kamal et al^14^	Cross-sectional	IL1β, TGFβ	e-cigarette smoker - conventional smoker - nonsmoker	86 Males	Cigarette: 14.7 ± 2.5 per day e-Cigarette: 10.1 ± 1.4 per day

###  General characteristics of the included studies

 The outcomes of the studies are presented in Supplementary file 2. The included studies were published between 2018 and 2022, and the number of patients enrolled in the studies ranged between 24 and 100. The total number of patients who participated in the seven studies was 563, with 431 men and 132 women.

###  Salivary biomarkers 

 In total, eleven biomarkers were assessed in seven case-control studies. The measured salivary biomarkers were IL-1β, IL-6, IL-8, IL-10, IL-1RA, CRP, TNF-α, PGE2, IL-15, IL-18, and TGF-β. Salivary IL-1β levels were measured in 6 studies. In three studies,^12-14^ it was higher in conventional smokers, and in others,^15-17^ e-cigarette users had higher levels of IL-1β. IL-6 biomarker was assessed in four studies, and all of them except one,^16^ reported higher levels in conventional smokers. Also, three studies evaluated IL-8 and^15-17^ showed higher IL-8 biomarker levels in conventional smokers, and in one study,^16^ it was vice versa.

 TGFβ and PGE2 levels were only reported in one study,^12,14^ and both these biomarkers were higher in conventional smokers. Two studies^15,17^ reported that CRP and IL-1RA levels in conventional smokers were higher than those in e-cigarette users, with higher IL-10 biomarker salivary levels in e-cigarette users. Regarding TNFα, two studies showed higher salivary levels in conventional smokers^15,17^ and one study reported vice versa.^16^ Finally, IL-15 and IL-18 salivary levels were assessed in one study^5^; this biomarker’s level was higher in conventional smokers.

 Due to the heterogeneity of the studies (I^2^ statistic > 90%), performing a meta-analysis was impossible. Even after a sub-group classification, homogenous data were not achieved.

## Discussion

 This systematic review compared the effect of conventional cigarettes and e-cigarettes on salivary biomarkers. Seven studies were finally included in this systematic review, and all were cross-sectional. The salivary biomarkers that were assessed showed different values between conventional and e-cigarette smokers.

 Levels of pro-inflammatory biomarkers, including IL-1β, IL-6, IL-8, CRP, TNF-α, IL-15, and IL-18, and anti-inflammatory biomarkers like TGF-β, IL-10, IL-1RA, and PGE2 were assessed in the included studies.

 Flieger et al^18^ investigated the levels of thiocyanate in the saliva of tobacco smokers in comparison to e-cigarette smokers and nonsmokers. Salivary thiocyanate is responsible for various neurological disorders (amblyopia, infant squint in children of smoking mothers) and endocrine diseases (an increase in the frequency of nodular goiter). They reported that the salivary thiocyanate levels in e-cigarette smokers were not significantly different from tobacco smokers but higher compared to nonsmokers. This finding suggests that e-cigarettes may not be as harmful as they were thought.

 Akiyama and Sherwood,^19^ in their systematic review in 2021 on changes in tobacco-related biomarker levels, concluded that using e-cigarettes could lead to a significant reduction in exposure to harmful substances compared to combusted cigarettes. In the present study, we specifically focused on salivary biomarkers and included several newly published studies.

 Interestingly, there were some conflicts in biomarkers’ measurements between studies, which made it difficult or even impossible in some cases to conclude the effect of conventional and e-cigarettes on salivary biomarkers. For instance, IL-1b was the most assessed biomarker in studies^12-17^ but half of them^12-14^ reported that its amount was higher in conventional smokers, and others showed that it was higher in e-cigarette users’ saliva. We hypothesize that these differences stem from heterogeneous methods in different studies. There were some critical differences in the survey of reasons why the outcomes of studies are not comparable. For instance, the use of antibiotics was not mentioned in the exclusion criteria in one study,^17^ while antibiotics might interfere with the quantity and quality of salivary biomarkers. Another issue was the different gender distribution in studies. There were two studies^13,14^ with only male participants. Another reason is that the time of cigarette and e-cigarette consumption in studies was not similar; thus, different exposure times might have led to various outcomes.

 Wadia et al^3^ assessed inflammatory cytokines (IL-1β and IL-8) in a group of established smokers before and after substituting vaping for smoking tobacco. They claimed that no definitive conclusions could be drawn from this dataset due to the limited sample size and large variations. Also, due to the study design of switching from tobacco smoking to vaping in participants, the results could be misinterpreted.

## Conclusion

 In this study, we could not agree on the different effects of conventional cigarettes and e-cigarettes on salivary biomarkers due to the heterogeneity of the included studies. We suggest that future studies use a standard method to enable more conclusive analyses.

## Competing Interests

 The authors declare that they have no financial and non-financial competing interests with regard to the publication of their work during submission.

## Consent for Publication

 Not applicable.

## Data Availability Statement


All the available data have been included in the submitted files, and Additional data are available in Supplementary file 1.

All the available data have been included in the submitted files, and Additional data are available in Supplementary file 2.

## Ethical Approval

 Not applicable.

## Funding

 No funding.
