# Supplementary material for: Effect of conventional cigarettes and e-cigarettes on salivary biomarkers: A systematic review
Source: J Adv Periodontol Implant Dent. 2024 Apr 22;16(1):44–8. doi: 10.34172/japid.2024.006 (PMC11252155; doi:10.34172/japid.2024.006)
Supplement: Supplementary file 1 — All the available data have been included in the submitted files, and Additional data are available in Supplementary file 1. [file japid-16-44-s001.pdf]

Dolatabadi et al, *J Adv Periodontol Implant Dent,* 2024, 16(1), 44-S1
doi: 10.34172/japid.2024.006
https://japid.tbzmed.ac.ir

Additional file 1. Search strategy

PubMed<br><br>((Electronic Nicotine Delivery System) OR (Electronic Cigarettes) OR (E-Cigs) OR (E Cigs) OR (E-Cig) OR (E Cig) OR (E-Cigarettes) OR (E Cigarettes) OR (E-Cigarette) OR (E Cigarette) OR (Electronic Cigarette) OR (Cigarette, Electronic) OR (Cigarettes, Electronic) OR (E-Cig) OR (ECig) OR (Vape) OR (Vapes) OR (Nicotine Vaping) OR (Nicotine Vapings) OR (Vaping, Nicotine) OR (Vapings, Nicotine) OR (Ecigarette) OR (Electronic Cigarette)) AND ((Smoking, Cigarette[Title/Abstract]) OR (Smoker[Title/Abstract]) OR (Smokers, Tobacco[Title/Abstract]) OR (Smoker, Tobacco[Title/Abstract]) OR (Tobacco Smoker[Title/Abstract]) OR (Tobacco Smokers[Title/Abstract]) OR (Nicotine[Title/Abstract])) AND ((Marker, Biological[Title/Abstract]) OR (Biological Marker[Title/Abstract]) OR (Biologic Marker[Title/Abstract]) OR (Marker, Biologic[Title/Abstract]) OR (Biological Markers[Title/Abstract]) OR (Biologic Markers[Title/Abstract]) OR (Markers, Biologic[Title/Abstract]) OR (Biomarker[Title/Abstract]) OR (Markers, Biological[Title/Abstract]) OR (Markers, Immunologic[Title/Abstract]) OR (Immune Markers[Title/Abstract]) OR (Markers, Immune[Title/Abstract]) OR (Marker, Immunologic[Title/Abstract]) OR (Immunologic Markers[Title/Abstract]) OR (Immune Marker[Title/Abstract]) OR (Marker, Immune[Title/Abstract]) OR (Immunologic Marker[Title/Abstract]) OR (Surrogate Endpoints[Title/Abstract]) OR (Endpoints, Surrogate[Title/Abstract]) OR (Surrogate End Point[Title/Abstract]) OR (End Point, Surrogate[Title/Abstract]) OR (Surrogate End Points[Title/Abstract]) OR (End Points, Surrogate[Title/Abstract]) OR (Surrogate Endpoint[Title/Abstract]) OR (Endpoint, Surrogate[Title/Abstract]) OR (Markers, Clinical[Title/Abstract]) OR (Clinical Markers[Title/Abstract]) OR (Clinical Marker[Title/Abstract]) OR (Marker, Clinical[Title/Abstract]) OR (Biochemical Marker[Title/Abstract]) OR (Markers, Biochemical[Title/Abstract]) OR (Marker, Biochemical[Title/Abstract]) OR (Biochemical Markers[Title/Abstract]) OR (Markers, Laboratory[Title/Abstract]) OR (Laboratory Markers[Title/Abstract]) OR (Laboratory Marker[Title/Abstract]) OR (Marker, Laboratory[Title/Abstract]) OR (Surrogate Markers[Title/Abstract]) OR (Markers, Surrogate[Title/Abstract]) OR (Marker, Surrogate[Title/Abstract]) OR (Surrogate Marker[Title/Abstract]) OR (saliva[Title/Abstract]))
Embase<br><br>**'electronic nicotine delivery system'**:ab,ti OR **'electronic cigarettes'**:ab,ti OR **'e cigs'**:ab,ti OR **'e cigarettes'**:ab,ti OR **'e cigarette'**:ab,ti OR **'cigarette, electronic'**:ab,ti OR **'cigarettes, electronic'**:ab,ti OR **'e cig'**:ab,ti OR **ecig**:ab,ti OR **vape**:ab,ti OR **vapes**:ab,ti OR **'nicotine vaping'**:ab,ti OR **'nicotine vapings'**:ab,ti OR **'vaping, nicotine'**:ab,ti OR **'vapings, nicotine'**:ab,ti OR **ecigarette**:ab,ti OR **'electronic cigarette'**:ab,ti

AND<br><br>**'smoking, cigarette'**:ab,ti OR **smoker**:ab,ti OR **'smokers, tobacco'**:ab,ti OR **'smoker, tobacco'**:ab,ti OR **'tobacco smoker'**:ab,ti OR **'tobacco smokers'**:ab,ti OR **nicotine**:ab,ti<br><br>AND<br><br>**'marker, biological'**:ab,ti OR **'biological marker'**:ab,ti OR **'biologic marker'**:ab,ti OR **'marker, biologic'**:ab,ti OR **'biological markers'**:ab,ti OR **'biologic markers'**:ab,ti OR **'markers, biologic'**:ab,ti OR **biomarker**:ab,ti OR **'markers, biological'**:ab,ti OR **'markers, immunologic'**:ab,ti OR **'immune markers'**:ab,ti OR **'markers, immune'**:ab,ti OR **'marker, immunologic'**:ab,ti OR **'immunologic markers'**:ab,ti OR **'immune marker'**:ab,ti OR **'marker, immune'**:ab,ti OR **'immunologic marker'**:ab,ti OR **'surrogate endpoints'**:ab,ti OR **'endpoints, surrogate'**:ab,ti OR **'surrogate end point'**:ab,ti OR **'end point, surrogate'**:ab,ti OR **'surrogate end points'**:ab,ti OR **'end points, surrogate'**:ab,ti OR **'surrogate endpoint'**:ab,ti OR **'endpoint, surrogate'**:ab,ti OR **'markers, clinical'**:ab,ti OR **'clinical markers'**:ab,ti OR **'clinical marker'**:ab,ti OR **'marker, clinical'**:ab,ti OR **'biochemical marker'**:ab,ti OR **'markers, biochemical'**:ab,ti OR **'marker, biochemical'**:ab,ti OR **'biochemical markers'**:ab,ti OR **'markers, laboratory'**:ab,ti OR **'laboratory markers'**:ab,ti OR **'laboratory marker'**:ab,ti OR **'marker, laboratory'**:ab,ti OR **'surrogate markers'**:ab,ti OR **'markers, surrogate'**:ab,ti OR **'marker, surrogate'**:ab,ti OR **'surrogate marker'**:ab,ti OR **saliva**:ab,ti
Scopus<br><br>(TITLE-ABS-KEY ((electronic AND nicotine AND delivery AND system) OR (electronic AND cigarettes) OR (e-cigs) OR (e AND cigs) OR (e-cig) OR (e AND cig) OR (e-cigarettes) OR (e AND cigarettes) OR (e-cigarette) OR (e AND cigarette) OR (electronic AND cigarette) OR (cigarette, AND electronic) OR (cigarettes, AND electronic) OR (e-cig) OR (ecig) OR (vape) OR (vapes) OR (nicotine AND vaping) OR (nicotine AND vapings) OR (vaping, AND nicotine) OR (vapings, AND nicotine) OR (ecigarette) OR (electronic AND cigarette))) AND (TITLE-ABS-KEY ((smoking, AND cigarette) OR (smoker) OR (smokers, AND tobacco) OR (smoker, AND tobacco) OR (tobacco AND smoker) OR (tobacco AND smokers) OR (nicotine))) AND (TITLE-ABS-KEY ((marker, AND biological) OR (biological AND marker) OR (biologic AND marker) OR (marker, AND biologic) OR (biological AND markers) OR (biologic AND markers) OR (markers, AND biologic) OR (biomarker) OR (markers, AND biological) OR (markers, AND immunologic) OR (immune AND markers) OR (markers, AND immune) OR (marker, AND immunologic) OR (immunologic AND markers) OR (immune AND marker) OR (marker, AND immune) OR (immunologic AND marker) OR (surrogate AND endpoints) OR (endpoints, AND surrogate) OR (surrogate AND end AND point) OR (end AND point, AND surrogate) OR (surrogate AND end AND points) OR (end AND points, AND surrogate) OR (surrogate AND endpoint) OR (endpoint, AND surrogate) OR (markers, AND clinical) OR (clinical

AND markers) OR (clinical AND marker) OR (marker, AND clinical) OR (biochemical AND marker) OR (markers, AND biochemical) OR (marker, AND biochemical) OR (biochemical AND markers) OR (markers, AND laboratory) OR (laboratory AND markers) OR (laboratory AND marker) OR (marker, AND laboratory) OR (surrogate AND markers) OR (markers, AND surrogate) OR (marker, AND surrogate) OR (surrogate AND marker) AND (saliva)))
