# Supplementary material for: Effect of conventional cigarettes and e-cigarettes on salivary biomarkers: A systematic review
Source: J Adv Periodontol Implant Dent. 2024 Apr 22;16(1):44–8. doi: 10.34172/japid.2024.006 (PMC11252155; doi:10.34172/japid.2024.006)
Supplement: Supplementary file 2 — All the available data have been included in the submitted files, and Additional data are available in Supplementary file 2. [file japid-16-44-s002.pdf]

Dolatabadi et al, *J Adv Periodontol Implant Dent,* 2024, 16(1), 44-S2
doi: 10.34172/japid.2024.006
https://japid.tbzmed.ac.ir

Additional file 2. Outcomes of studies

Study	Outcome
Ye et al.2018	e-cigarette smoker:<br>PGE-2 (pg/ml):344.19±172.19<br>IL-1β (pg/ml):182.31±100.90<br>cigarette smoker:<br>PGE-2 (pg/ml):588.72±207.87<br>IL-1β (pg/ml):350.69±364.96
Verma et al. 2021	e-cigarette smoker:<br>IL1 β (pg/ml):2.82±1.53<br>IL6(pg/ml):1.66±1.46<br>IL8(pg/ml):1.25±0.78<br>IL10(pg/ml):26.42±24.82<br>IL1RA(pg/ml):217.37±82.11<br>CRP(pg/ml):7238.13±4863.69<br>TNFα (pg/ml):30.39±5.67<br>cigarette smoker:<br>IL1β (pg/ml):1.41±1.52<br>IL6(pg/ml):2.02±1.69<br>IL8(pg/ml):1.19±0.59<br>IL10(pg/ml):30.60±30.33<br>IL1RA(pg/ml):245.17±92.34<br>CRP(pg/ml):7310.08±4526.14<br>TNFα (pg/ml):11.34±3.17
Miluna et al. 2022	e-cigarette smoker:<br>IL-6 (pg/ml): 107.39±92.37<br>IL-1β (pg/ml): 78.71±70.92<br>IL-8 (pg/ml): 445.09±135.12<br>TNFα (pg/ml): 66.60±181.63<br>cigarette smoker:<br>IL-6 (pg/ml): 49.69±94.59<br>IL-1β (pg/ml): 59.00±82.17<br>IL-8 (pg/ml): 355.52±120.67<br>TNFα (pg/ml): 66.59±92.44
Mokeem et al. 2018	e-cigarette smoker:<br>IL-1β (pg/ml):21.212±3.94<br>IL-6 (pg/ml):16.466±1.99<br>cigarette smoker:<br>IL-1β (pg/ml):115.758±7.57

	IL-6 (pg/ml):104.283±3.74
Faridoun et al. 2021	e-cigarette smoker:<br>IL1β (pg/ml): 2.84±1.51<br>IL6(pg/ml): 1.68±1.48<br>IL8(pg/ml): 1.27±0.80<br>IL10(pg/ml): 28.44±28.80<br>IL1RA(pg/ml): 215.39±86.09<br>CRP(pg/ml): 7236.15±4867.67<br>TNFα (pg/ml): 28.41±19.17<br>cigarette smoker:<br>IL1β (pg/ml): 1.39±1.54<br>IL6(pg/ml): 1.81±1.71<br>IL8(pg/ml): 1.28±0.67<br>IL10(pg/ml): 23.15±34.31<br>IL1RA(pg/ml): 245.58±96.32<br>CRP(pg/ml): 7308.10±3486.62<br>TNFα (pg/ml): 30.42±4.15
Ali et al.2022	e-cigarette smoker:<br>IL15 (pg/ml): 174.7±19.2<br>IL18 (pg/ml): 2793.1±196.4<br>cigarette smoker:<br>IL15 (pg/ml): 189.5±26.7<br>IL18 (pg/ml): 2869.8±285.6
Kamal et al.2022	e-cigarette smoker:<br>IL-1β (pg/ml): 10.58016<br>TGF-β (pg/ml): 36.75000<br>cigarette smoker:<br>IL-1β (pg/ml): 27.12906<br>TGF-β (pg/ml): 131.22308
